# About connections

**DOI:** 10.3389/fnana.2015.00061

**Published:** 2015-05-20

**Authors:** Kathleen S. Rockland

**Affiliations:** ^1^Department of Anatomy and Neurobiology, Boston University School of MedicineBoston, MA, USA; ^2^Cold Spring Harbor Laboratory, Cold Spring HarborNY, USA

**Keywords:** cerebral cortex, connectome, hierarchy, network, projections, reciprocal, topography

## Abstract

Despite the attention attracted by “connectomics”, one can lose sight of the very real questions concerning “What are connections?” In the neuroimaging community, “structural” connectivity is ground truth and underlying constraint on “functional” or “effective” connectivity. It is referenced to underlying anatomy; but, as increasingly remarked, there is a large gap between the wealth of human brain mapping and the relatively scant data on actual anatomical connectivity. Moreover, connections have typically been discussed as “pairwise”, point *x* projecting to point *y* (or: to points *y* and *z*), or more recently, in graph theoretical terms, as “nodes” or regions and the interconnecting “edges”. This is a convenient shorthand, but tends not to capture the richness and nuance of basic anatomical properties as identified in the classic tradition of tracer studies. The present short review accordingly revisits connectional weights, heterogeneity, reciprocity, topography, and hierarchical organization, drawing on concrete examples. The emphasis is on presynaptic long-distance connections, motivated by the intention to probe current assumptions and promote discussions about further progress and synthesis.

## Introduction

This mini-Review is a thumbnail treatment of some basic properties associated with long-distance cortical connections, mainly from the presynaptic perspective. This falls within the emerging “mesoscale” light microscopic framework, and largely leaves aside more detailed pre- and postsynaptic microcircuitry. I hope by discussion and specific examples to raise questions that might help in thinking about connections and in critically navigating the anatomical literature. Species and area differences, and interneuron distribution and pyramidal cell features (e.g., Elston et al., 2011), are largely neglected, owing to space constraints.

The early modern period of connectivity studies is often dated from the 1970’s, when tracer techniques using physiological axonal transport became routinely available (Köbbert et al., 2000; Lanciego and Wouterlood, 2011). Despite impressive progress in mapping the general connectivity of the brain over the intervening 45 years or so, there are still woefully few hard data, especially for the human cerebral cortex. Moreover, the relation between structure and function is often difficult to ascertain.

**Figure 1:**
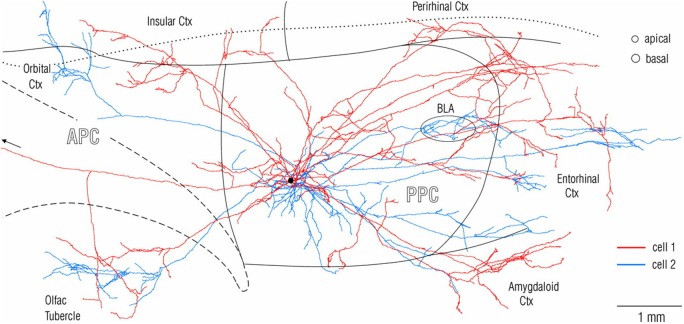
**Visualization at single-axon resolution, after intercellular fills, of two adjacent neurons (red and blue) in rat posterior piriform cortex**. Note widespread, but non-identical divergence of the two axons. Reproduced with permission from Johnson et al. (2000) Figure 2.

Experimental investigations of anatomical connections, in animal models, routinely begin with *in vivo* injection of tracer which is transported from the injected site, anterogradely to visualize all recipient (target) structures or retrogradely to visualize all input (source) structures. This unfortunately gives rise to a “source and target”, input/output convention, and is really shorthand for a much more complex reality. Axons of single neurons branch divergently to hundreds or thousands of postsynaptic neurons, and postsynaptic neurons can receive thousands of presynaptic inputs, converging from multiple different structures. Connectional divergence can be investigated by intracellular or very small extracellular injections of anterograde tracers, that produce stunning detail of an individual neuron, its axon branches and trajectory, and the distal terminations (Figure 1). This technique, despite the advantages of being high resolution, has suffered from the technical challenge of collecting strict serial histology sections and reconstructing intricate axonal branches in 3-dimensional brain space. In this regard, rapid developments in global imaging, as in the various “CLARITY” protocols, potentially offer a useful complementary approach (for applications and limitations, see, among others, Chung and Deisseroth, 2013; Osten and Margrie, 2013; Silvestri et al., 2013; Renier et al., 2014).

“Connections” or connectivity, as used here, refers to both distal pre-synaptic arborization and network organization, and is synonymous with “projections” (but not “projectome”, as sometimes used narrowly in reference to white matter). Connectivity summaries often show connections as lines between two intercommunicating structures. This is a convenient convention, but tends to cloud important issues of connectional strength, heterogeneity, reciprocity, topography, and hierarchy, issues that are briefly discussed in this mini-review. Longer reviews have treated the promise and pitfalls of newer mapping techniques (Yook et al., 2013), and further discussed the need for closing the gap between current “connectome” maps and the “real underlying anatomy” (Budd and Kisvárday, 2012; Mesulam, 2012; Catani et al., 2013).

## Connectional Strength

Connectional “strength” is hard to establish. Anatomical approaches rely on density of retrogradely labeled neurons or density of anterogradely labeled synaptic terminations. This is a reasonable first approximation, but one that must be used with caution. One major issue is that retrogradely labeled neurons and anterogradely labeled terminations are usually not homogeneous. Aside from anatomical heterogeneity, substantial evidence points to time-varying correlated activity (Calhoun et al., 2014; Kopell et al., 2014; Roland et al., 2014). A second issue is individual variability between brains, as influenced by maturational and other factors (cf. Markov et al., 2014). This can be mitigated by replicating results in multiple brains, but, in primate and even rodent brains, is likely to remain a factor. A third issue, as summarized elsewhere (Glickfeld et al., 2014), is that synaptic efficacy depends on multiple factors, such as the specific inhibitory or excitatory postsynaptic targets, synaptic location on the postsynaptic dendrite, and distribution of ionotrophic or metabotrophic receptors.

Equating “dense” projections with strength or efficacy can easily lead to erroneous conclusions. A long-standing puzzle, for example, has been that thalamocortical projections to the primary sensory areas comprise only a small proportion of the synaptic input to layer 4 neurons, but nevertheless strongly excite their postsynaptic targets (Peters and Payne, 1993; Latawiec et al., 2000; da Costa and Martin, 2011). Size of postsynaptic density or number of synaptic vesicles could be one factor, but the median size of the thalamic synapse is only slightly larger than that of other synapses (in cat: da Costa and Martin, 2011). One recent *in vivo* study reports, for rat somatosensory cortex at least, direct measurements of synaptic strength showing that thalamocortical and corticocortical synapses are both weak. Thalamic synapses are sufficiently convergent (~90 thalamic neurons: 1 postsynaptic cortical neuron) and coincidentally active to exert an “outsized influence” (Schoonover et al., 2014).

A second example of how numbers can mislead is from corticothalamic projections (Lee and Sherman, 2011; Sherman and Guillery, 2013). These can be subdivided into two broad types, distinguished in part by having large or small synapses. Type 2, with large synapses, are considered “driving”, but they originate from a much smaller neuronal population of neurons (in layer 5) than the population (in layer 6) giving rise to the “modulatory” type 1 projections (estimated as 1:50 in monkey temporal areas, Rockland, 1996; and see revised terminology in Lee and Sherman, 2011). In this case, the difference in efficacy has been attributed both to synapse size and more proximal postsynaptic location of the type 2 projections. In somatosensory thalamus of rodents, the effect of cortical “driver” input appears to be influenced by other, near coincident “driving” brainstem activity, converging on the same thalamocortical neuron (Groh et al., 2014).

*In summary*, the number of labeled neurons in retrograde tracer experiments or density of anterogradely labeled terminations is a useful estimate of connectional “strength,” but strictly speaking is a density measurement. It cannot and should not be equated with efficacy.

## Connectional Heterogeneity and Subtypes

There are connectional subtypes. The major cortical connectional systems (corticothalamic, corticocortical, corticostriatal, corticocollicular, and others) are conveniently classified to a first approximation by features of the parent cell in the source structure (laminar location and dendritic morphology), and the terminations and arborization in the target structure. There has been less consideration of finer subtypes, largely due to paucity of criteria, but it is worth considering that the degree of heterogeneity within any connection might actually be high.

One criterion for finer subdivisions is the classification of pyramidal cells, the neurons giving rise to connections. A recent study in rat frontal cortex identified “more than 10” different pyramidal subtypes on the basis of dendritic morphology and firing properties (van Aerde and Feldmeyer, 2014). This relatively small number (10) is likely to become much larger when factoring in criteria from gene expression levels and distribution of ion channels and receptors.

Subdivisions can be established from the basic characteristics of the pyramidal cell extrinsic axon. In what also appears to be a high degree of diversity, these vary morphologically in spatial divergence, axon diameter, and number of terminal arbors and specializations. A survey focusing on axon diameters alone, from projection neurons in monkey parietal area, found that each cortical and subcortical projection consists of axons with different diameters (Innocenti et al., 2014, their Table 2). The authors suggest this implies an “extraordinary complexity of axonal pathways operating at different conduction speed and generating different conduction delays between brain sites.”

Connectional subtypes have too often been seen as anatomical dualities. This carries an assumption of a functional dichotomy, even though anatomical evidence actually points to greater diversification. Corticothalamic projections, as already noted, have been classified into two broad categories on the basis of multiple criteria, but these have been further differentiated into two classes of type 1 axons and four classes of type 2 axons, based on morphological features of axon diameter, axon branching, and density of terminal specializations (Kultas-Ilinsky et al., 2003). The implication is that there are “multiple modes” of corticothalamic communication “feeding into a variety of functionally different neuronal networks, with each processing specific information.”

Cortical “feedback” and “feedforward” connections are another striking example, where pronounced structural differences (in laminar origin and termination, and spatial divergence of the terminal axon arbors) have been construed as evidence for two subtypes (discussion in Rockland, 1997; Markov and Kennedy, 2013; Markov et al., 2014). For feedback connections, however, there are at least four criteria for further subdivisions. (1) There is typically a bistratified distribution of cells of origin, in the supra- and infragranular layers (respectively, layers 2, 3A and 6); (2) Investigations of the ventral visual pathway report that a subpopulation of feedback-projecting neurons in layer 6, but not those in layer 2 or 3, uses synaptic zinc, an activity-related neuromodulator (Ichinohe et al., 2010); (3) The supra- and infragranular projecting populations differ in dendritic morphology, synaptic inputs, and local axon collateralization; and (4) There are substantial differences in the terminal axon portions, with Anderson and Martin (2009) distinguishing three subtypes of feedback axons from area V2 to V1 on the basis of axon caliber and density of terminal specializations. Similarly, a study in the auditory cortex notes that both feedforward and feedback projections include multiple “strands” within the main bundle, that target neurons in different layers (Hackett et al., 2014).

Evidence for finer subtypes of feedforward cortical connections is more indirect, but still suggestive. Parent cells, located through the thickness of layer 3, are likely to be heterogeneous; and even projections from area V1 to area V2 originate from neurons in layer 5 as well as layer 3 (Kennedy and Bullier, 1985; Sincich et al., 2010). Axon diameters are not uniform. EM data for cortical connections from both V1 and V2 to MT/V5 show a diameter distribution from less than 1.0 μm to 3.5 μm, with the majority of profiles being about 1.5 μm (Anderson and Martin, 2002).

Pyramidal cell axons have intrinsic collaterals (i.e., near the parent soma and within the same cortical area). The pattern of intrinsic collaterals in relation to extrinsic targets is another criteria for subtypes. From analysis of local axon collaterals, correlated with differences in dendritic trees, Wiser and Callaway (1996) distinguished two broad classes of layer 6 neurons in area V1 of macaques, each with further subdivisions. Also, corticothalamic neurons in layer 5 have widespread collaterals in the deeper layers, while the local collaterals of corticothalamic neurons in layer 6 project to layer 4 and are less divergent (Ojima et al., 1992). A safe assumption is that this heterogeneity is widely typical of various connectional systems, and that it is indicative of functional heterogeneity within connections.

The number, spatial extent, and laminar distribution of intrinsic collaterals has yet to be codified, but can be an important classification criterion of pyramidal cells. Importantly, a recent intracellular labeling investigation reports substantial within-layer heterogeneity for local collaterals of superficial pyramidal neurons (Martin et al., 2014). For 33 pyramidal cells, the number of local collaterals ranged to about 9 (supple. materials in Martin et al.). Co-registration of bouton clusters with optically imaged orientation domains revealed that intrinsic collaterals of a single pyramidal cell targeted a variety of different orientation domains. From these two results, the authors conclude that “instead of treating the lateral connections as a single homogeneous network, the real clue to its structure and function may lie in its heterogeneity of connections… and it is this heterogeneity that needs to be explained.”

*In summary*, there is abundant evidence for intra-class connectional heterogeneity. This can be assumed to support a variegated functional repertoire.

## Connectional Reciprocity

At the source-to-target, area-to-area level, reciprocity is common but clearly not ubiquitous, and why only some connections are reciprocal has not been explained. A number of corticothalamic, corticoamygdala, and corticocortical connections are reciprocal at the area-to-area level, whereas corticostriatal, corticocollicular, and other cortico-subcortical connections are not. Even at the area level, there are intriguing differences. For example, the amygdala projects to cortical areas that extend throughout the ventral visual stream, including area V1; but there are no projections from area V1 back to the amygdala (macaque: Freese and Amaral, 2005). In the network of visual cortical connections, temporal areas project back to multiple areas, including V1, but do not receive projections from V1 (Rockland and Van Hoesen, 1994; Rockland et al., 1994). Hippocampal CA1 has reciprocal connections with entorhinal cortex, but there are only input (afferent) connections from parietal cortex (macaque: Rockland and Van Hoesen, 1999), and only output (efferent) connections to frontal cortex (macaque: Barbas and Blatt, 1995; Cavada et al., 2000; rat: Vertes, 2006). One interesting discussion about “reciprocity” is that this may be a secondary consequence of other factors. Reciprocity of corticothalamic projections, for example, has been discussed as a consequence of the branching patterns of different classes of prethalamic inputs (“rule of parity” proposed by Deschênes et al., 1998).

At the cell-to-cell level there are essentially no data *in vivo* about direct reciprocity. For long-distance cortical connections, a general rule has been that pyramidal cells target a mixed population of other pyramidal neurons and inhibitory neurons, in an approximate ratio of 80% to 20%. Cortical inhibitory neurons necessarily represent a non-reciprocal component. Moreover, continuing with the example of cortical connections, these typically have multiple spatially separated terminal arbors, each contacting dozens to hundreds of postsynaptic neurons. An unanswered question is whether the pyramidal neurons in all or only one of the postsynaptic foci send return projections to the parent cell and/or to its immediate neighbors.

*In summary*, reciprocity is potentially an important network property, but, despite the common invocation, has not been actually demonstrated at the cell-to-cell level, is under-investigated and likely over-assumed even at the inter-areal level.

## Topographic and Non-Topographic Connections

Topographically organized maps are an important principle of cortical organization, especially for the primary and early sensory cortices, where the cortical areas have an ordered relationship to retinotopic, cochleatopic, or somatosensory peripheral space. Even for these sensory areas, however, there are many examples of seemingly non-topographic or “distributed” processing. Projections that target layer 1, in particular, are widely divergent; and feedback cortical and thalamocortical connections, visualized at the single axon level, clearly extend widely in layer 1, including over different cortical areas. In addition, local intrinsic collaterals of pyramidal cells are often widespread: collaterals of layer 2 neurons in the tree shrew (Rockland et al., 1982), of layer 2 neurons in rat retrosplenial cortex (Kurotani et al., 2013), of layer 3 pyramidal neurons (Gilbert and Wiesel, 1983; Martin et al., 2014), and of layer 6 corticoclaustral neurons (Katz, 1987). The spatial distances extend well beyond the parent neuron, likely to a different part of a topographic map (and see Haber and Calzavara, 2009 for cortico-basal ganglia networks).

**Figure 2:**
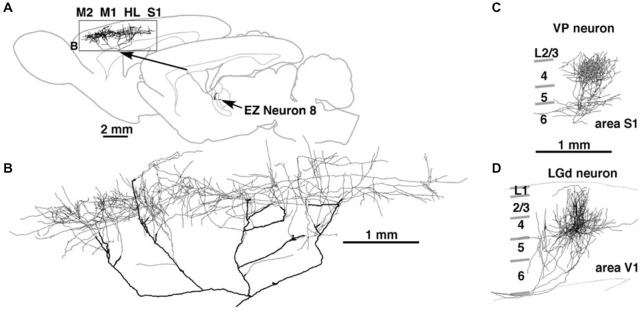
**Thalamocortical terminations at single axon resolution in rat motor cortex in (A) and, higher magnification, (B); in primary somatosensory cortex (C); and in primary visual cortex (D)**. Note high degree of divergence in motor cortex, and area-specific differences in tangential spread. Reproduced from Kaneko (2013) and, with permission, from figure 8 of Kuramoto et al. (2009). M1 = primary motor cortex; M2 = secondary motor cortex; HL = hindlimb representation; EZ = excitatory subcortical − input zone (of motor thalamus).

A single axon can have multiple arbors, in what might be a mixed topographic and non-topographic pattern. In rat motor cortex, a single thalamocortical axon to the middle layers spans more than 5.0 mm (Figure 2; Kaneko, 2013). In the early visual association areas in macaque, connections from V1 to MT/V5 and from V2 to V4 have three or four spatially separate arbors, distributed over 2.0–3.0 mm (Rockland, 1989, 1992). How the individual arbors relate to retinotopic organization in the target area is not known. Possibly, one of the arbors (“principal”?) may terminate in a topographically equivalent locus, while the others do not.

*In summary*, there is both topographic and non-topographic connectivity. This can be in relation to different systems (layer 1- vs. layer 4-terminating), different areas (motor vs. sensory), or different arbors of one axon in one area.

## Connections and Hierarchical Architecture

The identification of cortical areas has naturally led to a quest for how these are organized. A popular idea has favored a hierarchical organization, with primary sensory areas at the “lowest” level, initiating a serial connectivity (e.g., Felleman and Van Essen, 1991). There have, however, been extensive discussions about “reverse hierarchies” (i.e., connections directed *into* the sensory areas; Hochstein and Ahissar, 2002), and about “alternative hierarchies” based on properties such as reaction times (Petroni et al., 2001). Besides, the organization may be something quite different. One paper posits a mix of “partial gradients, fractures, swirls, regions that resemble separate areas in some ways but not others, and in not a lack of topographic maps but an excess of maps overlaid on each other, no one of which seems to be entirely correct” (Graziano and Aflalo, 2007). This evokes something like the “fractured topography” recognized for cerebellar cortex (Leergaard et al., 2006).

More specifically, one of several arguments against a purely sensory-based cortical hierarchy is the fact that primary sensory cortices are directly interconnected with multisensory cortices and with other primary and/or secondary sensory cortices (Rockland and Van Hoesen, 1994; Borra and Rockland, 2011; Stehberg et al., 2014). Physiological results corroborate that under certain behavioral conditions, primary sensory areas can be activated by other modalities (Henschke et al., 2015). In this regard, they are not just the “origin” or start-point of a unimodal sensory progression.

Another consideration is the increasing evidence for iterative, non-serial interareal interactions: (1) Simultaneous recordings from V1 and extrastriate area V4 in awake monkeys show that visual information about global contours in a cluttered background emerges initially in V4, ~40 ms sooner than in V1, and continues to develop in parallel in both areas (Chen et al., 2014). The anatomical interpretation is an incremental integration where feedback connections, in conjunction with local intrinsic connections, act to disambiguate signal from noise. (2) Re-examination of cortical processing streams in the visual cortex emphasizes an “expanded neural framework for processing object quality… containing neural representations of object quality both utilized and constrained by at least six distinct cortical and subcortical systems.” This is contrasted with the earlier view of the ventral visual pathway “as a largely serial staged hierarchy that culminates in singular object representations” (Kravitz et al., 2013).

*In summary*, hierarchical organization does not convincingly capture the full complexity of anatomical connectivity. The notion of a hierarchy does not incorporate subcortical loops and neglects the temporal dimension or dynamics, both of which are critical aspects of anatomical and functional organization.

## Concluding Remarks

What are connections? They are not arrows, not homogeneous, and, despite the popularity of graph theory (Bullmore and Sporns, 2009), are only partly approximated by “edges.” Anatomically depicted connections—whether in images or tables—can look static; but the idea of fixed anatomical connectivity is deceptive and an accident of methodology, just as musical notes are only indicators of the actual music in performance. The static mode of representation makes it easy to forget that connections are heterogeneous in efficacy, in type, and in time, and operate through flexible roles and flexible routes in different behaviors.

## Conflict of Interest Statement

The author declares that the research was conducted in the absence of any commercial or financial relationships that could be construed as a potential conflict of interest.
